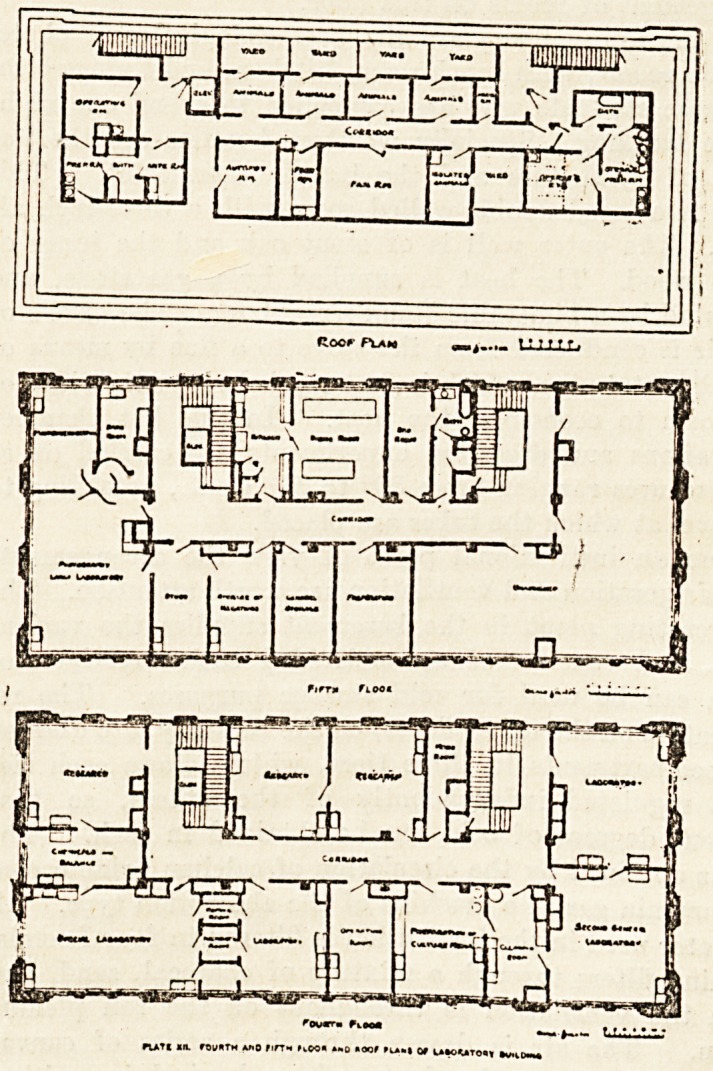# The Rockefeller Institute

**Published:** 1910-02-19

**Authors:** 


### THE ROCKEFELLER INSTITUTE.

### A MODEL RESEARCH LABORATORY.

(By our Special Correspondent.)

Early in 1907 a party of medical men met at the Arlington Hotel, in Washington, to consider the advisability of founding in America a special institute for the promotion of research in medicine. In Germany, France, Russia, Japan, and in England such institutions were already in existence. In the United States, although much research was being done at the laboratories attached to general and special hospitals, no separate institution could be found. With the help of Mr. Rockefeller's munificent donations it was possible to start and endow such a one, and the result of the deliberations at Washington was the foundation and formal incorporation, on June 14, 1901, of the Rockefeller Institute for Medical Research. A year later the entire property on which the present buildings stand, the block between East 66th and 67th Streets, was purchased, and building operations were started. The corner stone of the main building was laid two years later, and on May 11, 1906, the Institute was formally opened. Since then it has proved its usefulness by the mass of research work that has been done within its walls, and to-day it stands as one of the most interesting scientific monuments in New York City.

To the visitor inspecting the Institute for the first time, the passage from the noise and bustle of the riverside avenues to the quiet seclusion of the laboratories is striking. There is yet much to be done before the environment of the Institute can be regarded as ideal. Few streets are more filthy and reminiscent of Coleridge's libel on Cologne than the avenues that lead from East River, and at present, when building operations are in full swing on the property on the site of the adjunct hospital which is shortly to be erected alongside the Institute, the visitor who is familiar with the prim cleanliness of the Paris Pasteur Institute, the Berlin Charité buildings, and our own Lister Institute may be forgiven if the first view of the Rockefeller buildings creates an unfavourable im-

The Main Building, Rockefeller Institute.

pression. The buildings of yellow brick with limestone facings are by no means imposing, but they have an air of quiet dignity which goes far to remove the bad impression made upon the sensitive visitor's mind by the tramp through the litter-strewn avenues and the havoc which surrounds the Institute itself.

Plate XI. First, Second and Third Floor Plans of Laboratory Building.

Plate XII. Fourth and Fifth Floor and Roof Plans of Laboratory Building.

There are three distinct buildings: the laboratory proper, the animal house, and the power station—all built on an eminence overlooking the river on the one side and the city on the other. The laboratory consists of basement, five full stories, and a roof station, which serves as an animal hospital. In the basement are housed the pumps, ventilating filters, refrigeration and steam apparatus, the large centrifugees storage bins, and all the various heavier machinery that the workers constantly use. Here, too, are the carpenters' shops and the janitor's suite of rooms. On the first floor is a large and well-equipped library, an assembly room, and the Directors' board-room, besides rooms for the administrative staff. On the second floor is the large chemical laboratory, divided into two parts, the one serving as a general chemical laboratory, while the other is devoted to special experimental work. On this floor, too, are several rooms for various apparatus and machinery. In the mechanical room, for example, is a fine hydraulic pump for maintaining a high constant negative pressure for filtration purposes, the double-cylinder Geryk vacuum pump for maintaining a high constant negative pressure for alcohol and water.

On the third floor are the experimental pathological laboratories. Here the visitor comes upon an up-to-date, excellently fitted-up operating-room, with adjoining anæsthetic and preparation rooms, bath and drying rooms, all destined for the use of the animals used in research work. The bath is large enough to be used for goats or sheep, while the drying cages are specially designed to allow hot air to be blown through. Next to this suite of operating-rooms is the large general hospital for animals separated by a dividing corridor from the secretary's room. The plans show better than any description the general arrangement of rooms on this floor.

On the upper floors are the bacteriological and photographic rooms, with lunch-room, kitchen, and living-rooms for those students who are compelled to spend one night in the building. Specially worthy of notice are the fine series of incubators and the hot-air thermostat. This latter is a laced double-walled room with a three-inch air space. The outer wall is of stout oak and the inner of white wood. The heat is supplied by a gas stove, and regulated by a bimetallic Roux's regulator. The excess of hot air is conducted from the stove to a flue by means of a small metal pipe, which is first carried along the sides of the room to economise the heat. In this hot chamber cultivations and digestion experiments are carried on at temperatures ranging from 35° to 39° Cent., according to the level at which the tubes are placed.

From an institutional point of view the arrangements for refrigeration and ventilation are worth attention. The refrigerating plant in the basement supplies the various floors, and consists of an undivided entire refrigerator which can be used for cold storage purposes. The refrigerators on the upper floors, on the contrary, are divided into compartments in three tiers, which allows each tier to be regulated independently of the others, so that different degrees of cold can be obtained in each. The cold is obtained by the circulation of calcium brine cooled by ammonia gas in a machine of the absorption type. All the water used in the institution is filtered in four Loomis-Manning filters through a mixture of charcoal, sand, and shell; the ventilation is throughout on the fan plenum system. The air is drawn through a series of canvas bags, which collect the dust and mechanical impurities, passed over steam-heated coils by an electric blower in the basement, and finally blown into the various rooms, the intake being registered by adjustable registers. All foul air is removed by electric blowers placed on the roof.

Everything in the laboratory appears to have been designed for the comfort and convenience of the workers, and not a little for the convenience of the animals which are being used for research purposes. The animal hospital, indeed, is a model of its kind: we have seen general hospitals designed for human beings which are hardly comparable, from a hygienic point of view, to the fine wards provided for the Rockefeller dogs. Equally excellent is the special operating theatre on the roof, which can give points to the operating theatres in many general hospitals as far as light and facility for cleaning are concerned. Within the institution scrupulous cleanliness is the rule, and the absence of foul smells, dirty litter, and dust is remarkable when one considers the variety of work that is caried on every day.

Connected with the main building are the power house and the animal house. In the latter are the stables, where the horses and cattle for serum experiments are kept. The stalls here are neatly kept and as clean as can be expected. Altogether the institution from a structural and administrative point of view well repays a visit. We hope in a future issue to give a summary of the recent work that has been accomplished in it.

## Figures and Tables

**Figure f1:**
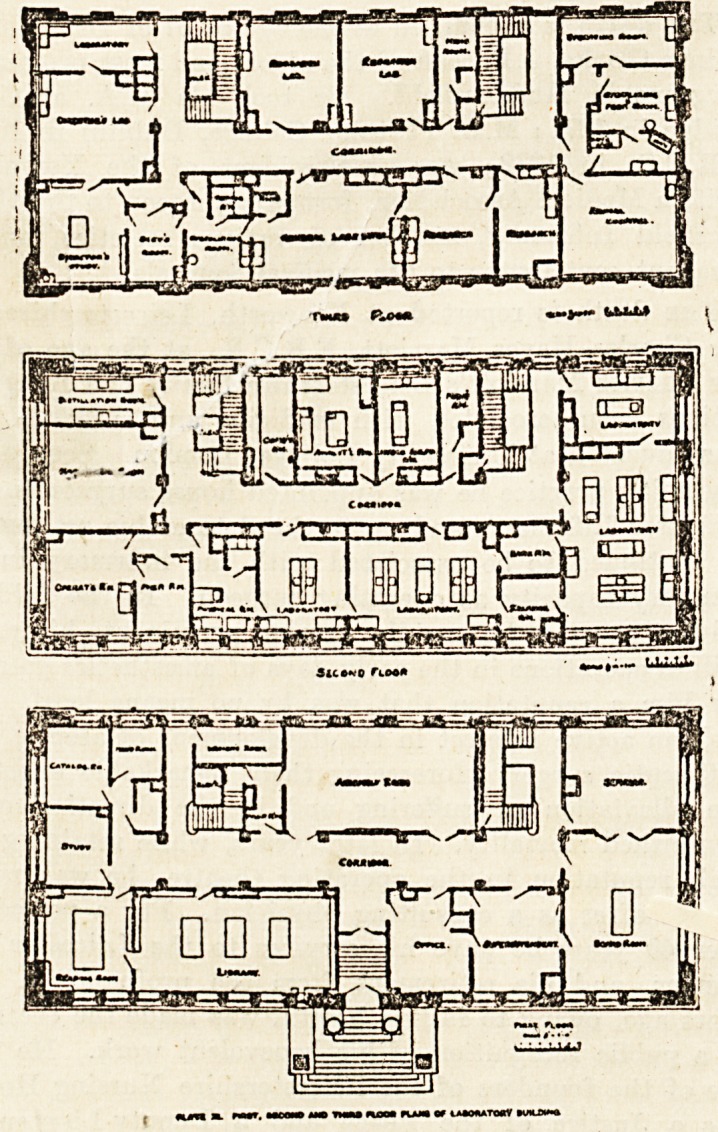


**Figure f2:**
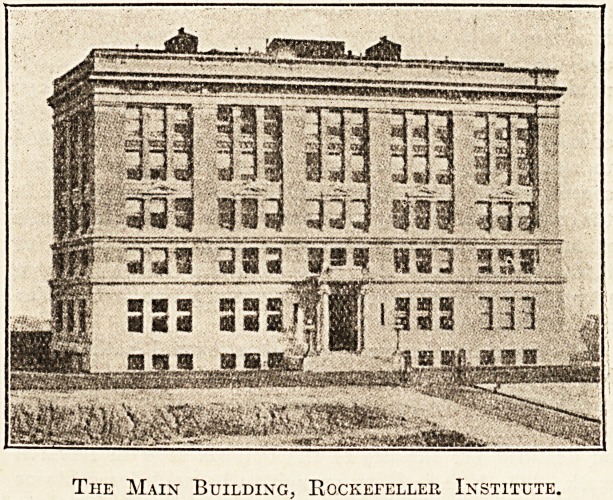


**Figure f3:**